# Glioblastoma, IDH-wildtype with primarily leptomeningeal localization diagnosed by nanopore sequencing of cell-free DNA from cerebrospinal fluid

**DOI:** 10.1007/s00401-024-02792-0

**Published:** 2024-09-03

**Authors:** Nik Sol, Evert-Jan Kooi, Marc Pagès-Gallego, Dieta Brandsma, Marianna Bugiani, Jeroen de Ridder, Pieter Wesseling, Carlo Vermeulen

**Affiliations:** 1 Netherlands Cancer Institute - Antoni Van Leeuwenhoek Hospital, Dept. of Neuro-Oncology, Amsterdam, The Netherlands; 2 Amsterdam University Medical Centers/VUmc, Dept. of Pathology, Amsterdam, The Netherlands; 3 Center for Molecular Medicine, Utrecht University Medical Center, Utrecht, The Netherlands; 4 Oncode Institute, Utrecht, The Netherlands; 5 Princess Máxima Center for Pediatric Oncology, Utrecht, The Netherlands

A 74-year old male presented with a headache, hand tremors, and gait and cognitive disturbances, which progressively worsened over the previous two months. He had a history of prostate cancer for which he was treated with radiotherapy 5 years earlier and a neuroendocrine tumor (NET) grade 1 in his terminal ileum 2 months prior. MRI of the brain revealed leptomeningeal contrast enhancement around the brainstem, cerebellum, and surrounding the cranial nerves in the absence of intraparenchymal lesions (Fig. 1a-c). The differential diagnosis at that time included a ‘neuroinflammatory disease’ and metastatic disease (also acknowledging that leptomeningeal metastasis from prostate cancer and grade 1 NET can occur [10]). Apart from the NET in the ileum, gallium-dotate and fluorodeoxyglucose (FDG) PET-CT scan revealed no extracranial primary tumor or metastatic disease. Cerebrospinal fluid (CSF) obtained by lumbar puncture showed a slightly increased leukocyte number (14 cells/µl), normal glucose (2.9 mmol/l), and an increased total protein levels (8.75 g/l), consistent with leptomeningeal metastatic disease. Cytology and circulating tumor cell analysis of CSF [8] remained negative, even at a third lumbar puncture. Next-generation sequencing of cell-free DNA (cfDNA), however, revealed a telomerase reverse transcriptase (*TERT*) promoter mutation, strengthening the hypothesis that the patient actually had a leptomeningeal malignancy [3].

**Fig. 1:**
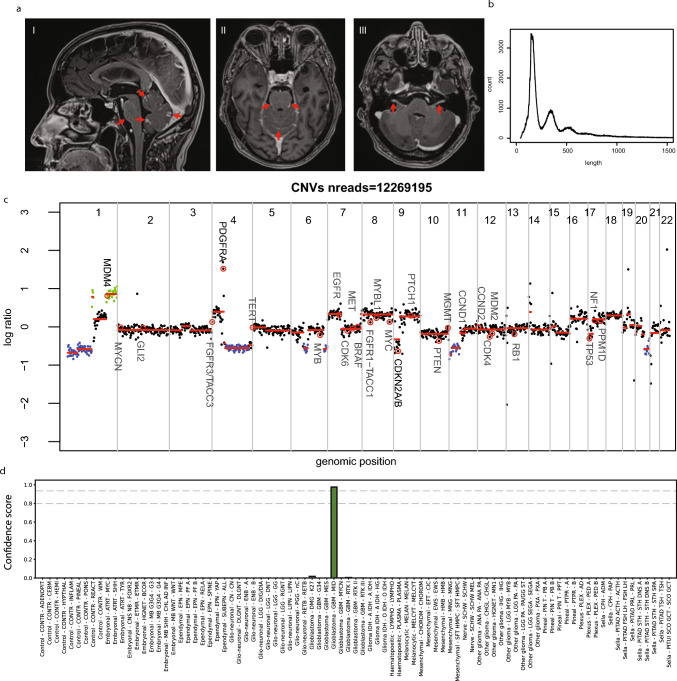
Clinical diagnostics. **a** Sagittal and axial post-contrast T1-weighted MRI images show contrast enhancement around the folia of the cerebellum and the pons (indicated arrows in I, II). Axial post-contrast T1-weighted image shows contrast enhancement of cranial nerve VII and VIII on both sides (arrows in III). **b** Size distribution of the sequence reads obtained from CSF DNA sequencing. **c** CNV profile obtained from nanopore sequencing. **d** Sturgeon classification with a confident (> 0.95) classification as Glioblastoma—MID

Recently, nanopore sequencing for cfDNA from CSF was reported [1], showing promising results in some but not all cases. As a biopsy was considered too burdensome and with a fair chance of not yielding a representative tumor sample, we opted for performing nanopore sequencing of CSF. Approximately 200 ng of DNA was extracted from 2 mL of CSF. Using an adjusted library prep protocol (see Supplementary information) sequencing of 100 ng of the sample resulted in 18 million reads. Mapped read lengths show the typical ~ 150 basepair periodicity expected for cfDNA (Fig. 1b), with the majority of fragments in the 143–175 basepair range. The copy-number variation (CNV) profile (Fig. 1c) revealed several alterations, including *PDGFRA* and *MDM4* gain and *CDKN2A/B* loss, the high deviation indicating a high tumor fraction. DNA methylation analysis using the Sturgeon classifier [9] on the full or subsampled dataset yielded a high confidence score for Glioblastoma, subtype Midline (GBM—MID) (Fig. 1d) (Supplementary Table 1). Later on, DNA methylation analysis using the Illumina Infinium EPIC methylation array and the Heidelberg classifier version 11b4 [2] confirmed the classification as GBM—MID (calibrated score > 0.99) as well as the alterations in the CNV profile. In contrast, sequencing DNA extracted from the cell pellet obtained from CSF yielded a flat copy-number profile and indicated with a high confidence ‘inflammatory tissue’, likely due to the predominance of inflammatory cells in the pellet (Supplementary Fig. 1).

Given the widespread intracranial disease and the clinical deterioration, best supportive care was provided. The patient's condition rapidly worsened and he soon succumbed. Autopsy revealed dispersed thickening and opacity of the leptomeninges especially around the frontal lobes (Fig. 2a), the cerebellum and brainstem. Macroscopy did not reveal a tumor mass within the brain parenchyma. Microscopically widespread infiltration of tumor cells throughout the leptomeninges was present (Fig. 2b). Intraparenchymal growth was subtly evident on microscopic evaluation showing diffuse infiltrative growth beneath the ependymal lining of the ventricles (Fig. 2c,d) and subpially in the mesencephalon (Supplementary Fig. 2). The tumor cells exhibited astroglial morphology and highly atypical nuclei with coarse chromatin (Fig. 2d). Immunohistochemistry confirmed the glial nature of the tumor (Olig2; Fig. 2e). Occasional mitotic figures were observed, but necrosis and florid microvascular proliferation were absent. Notably, tumor cell proliferation as evidenced by Ki-67 staining was moderate to high (~ 20–30%; Fig. 2f). These post-mortem findings in combination with identification of the *TERT* promoter mutation allowed for a histomolecular diagnosis of glioblastoma, IDH-wildtype [5], completely in line with the diagnosis suggested by nanopore sequencing of CSF during the life of the patient.

**Fig. 2:**
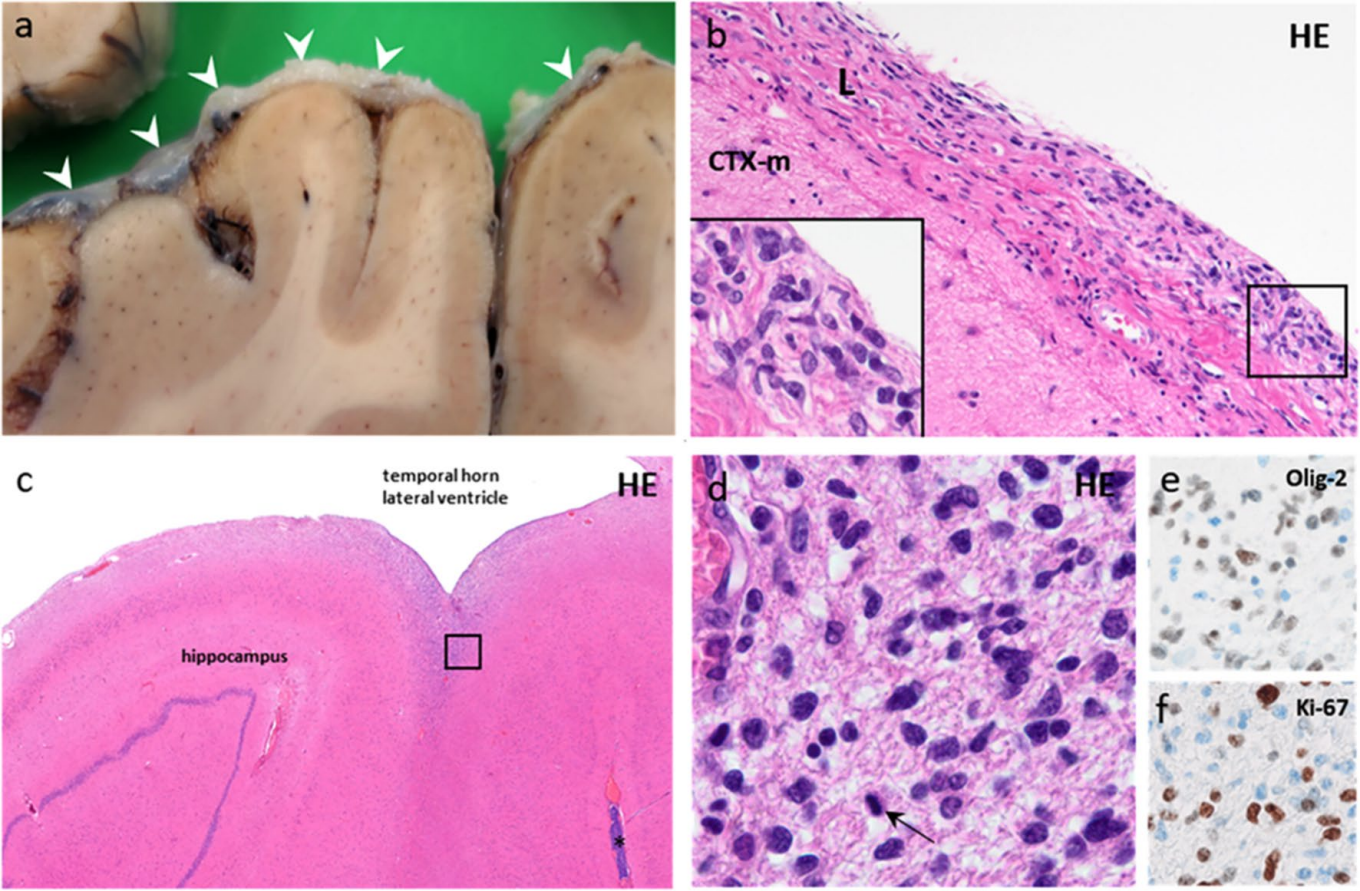
Autopsy findings. A coronal brain section showed leptomeningeal opacification and thickening (**a**; white arrowheads), the parasagittal part with a rough surface partly due to the presence of granulations of Pacchioni. Microscopically, atypical cells are diffusely spread throughout the leptomeningeal compartment (**b**). In several regions, such cells were also (but relatively subtly) present in the subependymal/periventricular region, e.g., near the hippocampus (**c**, **d**; square box), with more dense accumulation of these cells in an adjacent, deeply invaginating cerebral sulcus (*). Immunohistochemical staining for Olig2 (**e**) and Ki-67 (**f**) confirmed the glial nature of the tumor and its moderate proliferative activity, respectively. *L*: leptomeninges; *CTX*: molecular layer of the cerebral cortex; *HE*: hematoxylin and eosin staining

In conclusion, we present a case of a glioblastoma, IDH-wildtype, growing primarily in the leptomeninges. Such cases have rarely been reported before [4, 6, 7] and are very challenging to diagnose. DNA methylation profiling using nanopore sequencing of cfDNA from CSF and the Sturgeon classifier offered in this case a fast and minimally invasive means to reach a straightforward suggestion for a diagnosis. We hypothesize that the high amount of tumor DNA recovered from CSF is a result of the large contact surface between tumor cells and the CSF. In small and circumscribed tumors, cfDNA recovery and sequencing is likely more challenging. The finding of a *TERT* promoter mutation, the alterations in the CNV profile, and a high confidence classification gave sufficient evidence for the diagnosis high-grade glioma/glioblastoma. This diagnosis was very helpful for the patient and his family, as it served as the basis to continue with palliative care.

## Supplementary Information

Below is the link to the electronic supplementary material.

Supplementary file1 Cell pellet sequencing. The cell pellet obtained from the same CSF used for cfDNA sequencing was also processed and sequenced. a. Size distribution density plot of the sequence read length, showing a much larger read size than in the cfDNA. b. Copy-number profile, showing a flat profile with few clear variations. c. Sturgeon result showing a high confidence score for inflammatory tissue. (PDF 1493 KB)

Supplementary file2 Autopsy findings continued. Examination of the mesencephalon at the level of the substantia nigra (SN) revealed no clear macroscopic abnormalities (a). Microscopic examination (b; black arrows) revealed the presence of glioma subpially along the outer contour of the mesencephalon (c; high power image corresponding to the square box with dotted line indicated in b) as well as in the leptomeninges (not shown). Additionally, tumor growth was observed in the leptomeningeal compartment surrounding the oculomotor nerve (n. III) (d; high power image corresponding to the uninterrupted lined box square in b). Abbreviations: n.III: oculomotor nerve; HE: hematoxylin and eosin (H&E) staining; SN: Substantia nigra (PDF 6301 KB)

## Data Availability

Data is available upon reasonable request.
