# Supplementary figures and images for: Glioblastoma, IDH-wildtype with primarily leptomeningeal localization diagnosed by nanopore sequencing of cell-free DNA from cerebrospinal fluid
Source: Acta Neuropathol. 2024 Sep 3;148(1):35. doi: 10.1007/s00401-024-02792-0 (PMC11371860; doi:10.1007/s00401-024-02792-0)

## Supplementary figure 1

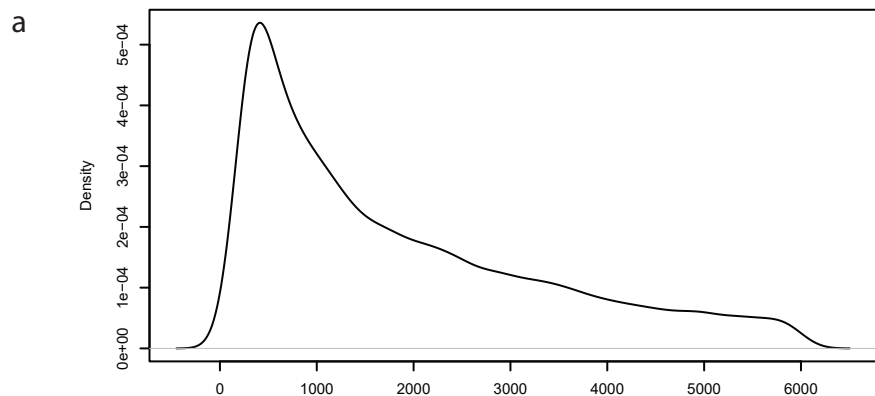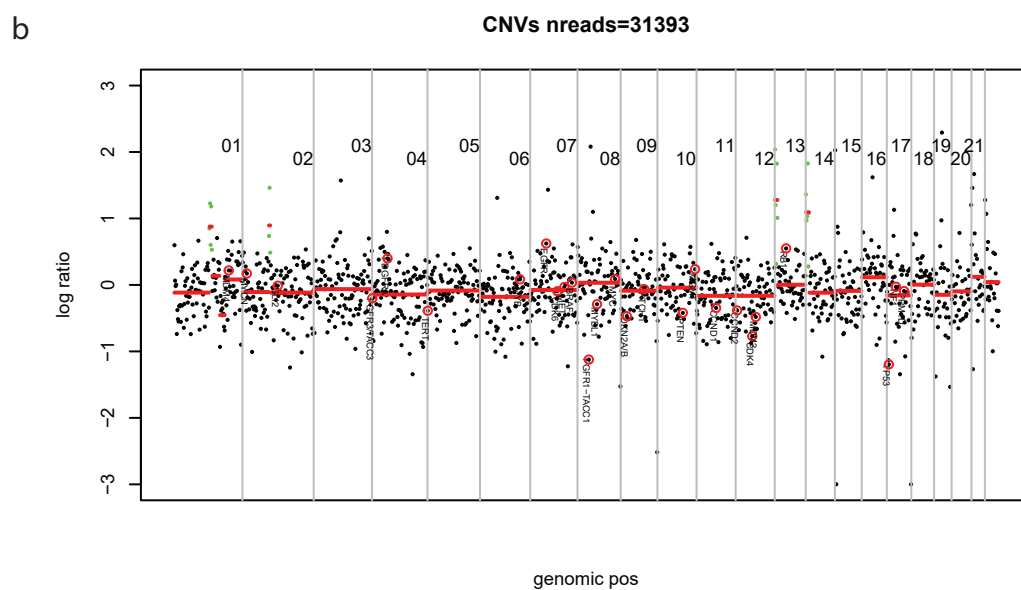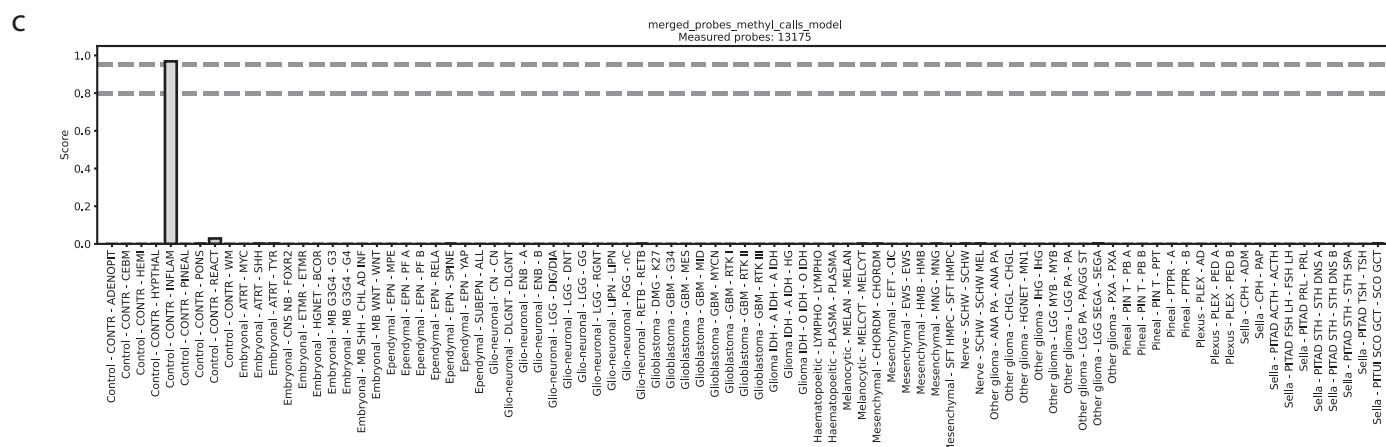

Supplement: Supplementary file 1 — Supplementary file1 Cell pellet sequencing. The cell pellet obtained from the same CSF used for cfDNA sequencing was also processed and sequenced. a. Size distribution density plot of the sequence read length, showing a much larger read size than in the cfDNA. b. Copy-number profile, showing a flat profile with few clear variations. c. Sturgeon result showing a high confidence score for inflammatory tissue. (PDF 1493 KB) [file 401_2024_2792_MOESM1_ESM.pdf]

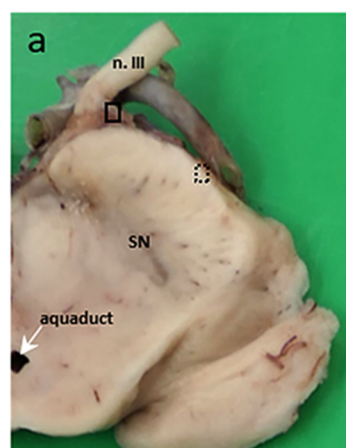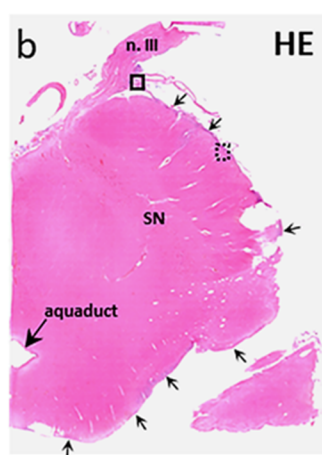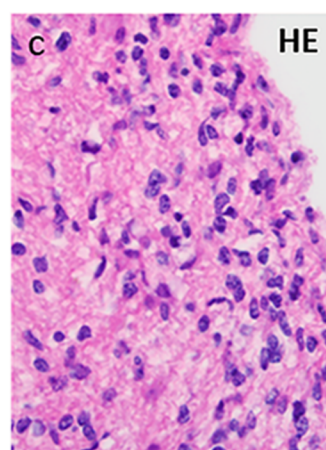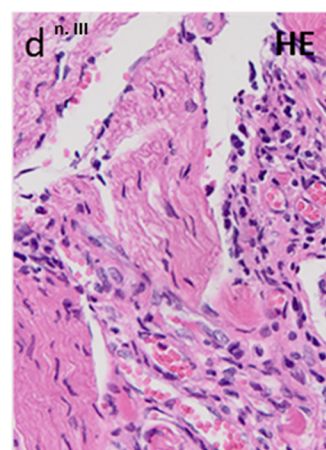

Supplement: Supplementary file 2 — Supplementary file2 Autopsy findings continued. Examination of the mesencephalon at the level of the substantia nigra (SN) revealed no clear macroscopic abnormalities (a). Microscopic examination (b; black arrows) revealed the presence of glioma subpially along the outer contour of the mesencephalon (c; high power image corresponding to the square box with dotted line indicated in b) as well as in the leptomeninges (not shown). Additionally, tumor growth was observed in the leptomeningeal compartment surrounding the oculomotor nerve (n. III) (d; high power image corresponding to the uninterrupted lined box square in b). Abbreviations: n.III: oculomotor nerve; HE: hematoxylin and eosin (H&E) staining; SN: Substantia nigra (PDF 6301 KB) [file 401_2024_2792_MOESM2_ESM.pdf]
